# Wild boar carcasses in the center of boar activity: crucial risks of ASF transmission

**DOI:** 10.3389/fvets.2024.1497361

**Published:** 2024-12-19

**Authors:** Jan Cukor, Monika Faltusová, Zdeněk Vacek, Rostislav Linda, Vlastimil Skoták, Petr Václavek, Miloš Ježek, Martin Šálek, František Havránek

**Affiliations:** ^1^Forestry and Game Management Research Institute, Jíloviště, Czechia; ^2^Faculty of Forestry and Wood Sciences, Czech University of Life Sciences Prague, Prague, Czechia; ^3^Faculty of Forestry and Wood Technology, Mendel University Brno, Brno, Czechia; ^4^State Veterinary Institute Jihlava, Jihlava, Czechia; ^5^Czech Academy of Sciences, Institute of Vertebrate Biology, Brno, Czechia; ^6^Faculty of Environmental Sciences, Czech University of Life Sciences Prague, Prague, Czechia

**Keywords:** African swine fever, disease control, biosecurity, wild boar behavior, camera-trapping

## Abstract

African swine fever (ASF) is a highly virulent disease rapidly spreading through Europe with fatal consequences for wild boar and domestic pigs. Understanding pathogen transmission among individuals and populations is crucial for disease control. However, the carcass attractiveness for boars was surprisingly almost unstudied. Here, we evaluated if the wild boar carcasses are perceived as an attractant compared to the control sites throughout the year. For this purpose, 28 wild boar carcasses were placed in seven forest stands and continuously monitored in 2019–2020 by camera traps combined with control locations situated at least 200 m away in comparable habitats. Overall, we have recorded 3,602 wild boar visits, from which 3,017 (83.8%) were recorded in locations with placed carcasses and 585 (16.2%) in control locations. Most visits were recorded after sunset and before sunrise, corresponding to common peaks of wild boar activity. On average, the first visits were detected 4.7 days after carcass placement. Contrarily, it was 61.5 days for the control site. In conclusion, we have proven an enormous wild boar carcass attractiveness for boars, which exhibits an entirely new aspect of wild boar behavior. Therefore, the carcass removal is a crucial measure for controlling the spread of ASF.

## Introduction

African swine fever (ASF) is a global viral disease affecting wild boar (*Sus scrofa* L.) and domestic pigs (*Sus scrofa domesticus* Erxleben) with a negative socioeconomic impact, especially on the pork industry (1–3). From 2007, when ASF was detected in Eastern Europe, the virus had rapidly spread to numerous Central and Western European countries, including Germany, Slovakia, Poland, Czech Republic, and Italy (4–6). Moreover, the ASF is also spreading throughout Asia, including China and other southeastern regions. In the worst-case scenario, the global effects of ASF disease on food security can increase the number of humans at risk of hunger by 13–14 million, especially in India and Southeast Asia (7). Therefore, controlling the spread of ASF in the wild boar population is one of the crucial topics worldwide, not only in Europe.

Infections with virulent strains of ASFV often lead to fatal disease in *Suidae* individuals. Wild boars or domestic pigs infected with virulent strains of ASFV usually die up to 10 days after infection, and with the genotype II the mortality rate reaches 90% or even more (8, 9). In the acute-lethal course of ASF, most animals die within 7 to 14 days after infection (10, 11). However, previous evidence suggests that some animals may survive longer or completely recover (12). However, seropositive animals, which theoretically could spread the virus, are rather exceptional in the wild boar population and thus do not play an epidemiological role regarding virus perpetuation (13). In domestic pigs, ASFV transmission by survivor pigs was observed in one study (14), whereas another study showed no transmission (15) over the entire in-contact phase from survivors to sentinels during infections with moderately virulent virus strains. The spreading of the ASF virus differs according to conditions in the area of the virus occurrence. The sylvatic cycle, tick-pig cycle, and domestic cycle are described for the sub-Saharan Africa region (16, 17). The situation is unlike Europe, where most outbreaks were found in wild boar populations (5, 16). Since 2007, ca. 50,000 cases of ASF have been reported in Europe, and the vast majority (86%) were confirmed in wild boar (18). Based on differing European climates and environments in comparison to sub-Saharan Africa, the new epidemiologic cycle of wild boar habitat was defined. The wild boar habitat cycle is characterized by direct transmission between infected and susceptible wild boar and indirect transmission through carcasses and contaminated environment (19).

The possible ways of ASF transmission through infected carcasses were described by Probst et al. (20). The risky behavior of wild boar towards infected carcasses consisted of direct contacts especially by sniffing and poking on the carcass and much less by chewing bare bone once skeletonization of the carcasses was complete which was most frequently documented for piglets (20). Moreover, wild boar cannibalism was initially detected in another study during the winter when the carcass biomass is stable and preserved by low temperatures (21). This behavior represents a very effective way of infection transmission. On the other hand, it seems that in hot, semiarid climate conditions, the carcass decomposes rapidly reducing opportunity for live wild pigs to interact with carcass compared to milder climates in Central Europe (22).

The risk of ASF transmission through carcasses is significant due to the relatively long-term virus stability. The long-term survival of the virus in the environment depends on several environmental and climatic factors, with temperature as one of the most important (23). The ASF virus can survive for over a year in the blood at 4°C, several months in boned meat, and several years in frozen carcasses (24, 25). Moreover, ASF virus can persist in contaminated soils where the virus stability depends on the soil type, pH, organic material percentage, and to a lesser extent, the ambient temperature (26, 27). The low temperatures are crucial in the process of overwintering when the virus can persist in the carcass from the autumn through winter with the following risk of cannibalism of infected body mass in spring, which could result in the subsequent ASF outbreaks in the wild boar population (21).

Based on the abovementioned findings, it is evident that the infected carcasses play a critical role in ASF transmission in the wild boar population. This leads to various biosecurity measures including carcass disinfection (28, 29) or removal of carcasses from infected areas (30, 31). Surprisingly, there is still insufficient evidence describing the attractiveness of the wild boar carcass for their fellow boar, which may be a crucial behavioral aspect for setting effective disease control strategies. Therefore, the main aims of this study were to (i) describe the attractiveness of wild boar carcass for individuals; (ii) evaluate the sex and age structure of individuals in the location with a carcass and the control site; and (iii) evaluate the effect of daytime and season on visit intensity of the carcass compared to the control site on randomly chosen locations in comparable habitat.

## Methods

### Data acquisition

The research was conducted in seven forest stands in the Czech Republic, Central Europe. The selected sites were previously described by Cukor et al. (21), and this research builds on the data collected during that study by placing additional carcasses on sites in the subsequent seasons. The forests mainly consisted of Norway spruce (*Picea abies* [L.] Karst.) with young forest stands (39 years on average) in altitudes ranging from 358 to 626 m a.s.l. (see Table 1). The study sites have humid continental and oceanic climates, characterized by warm to hot summers and cold winters, and, respectively, by cool summers and mild winters with a relatively narrow annual temperature range (32). The population density of wild boar is comparable among individual selected sites (Cukor, unpublished data). All of the study sites are located in the Czech Republic, that has one of the highest wild boar population densities (1.15 to 5.31 ind./100 ha) in Central Europe (33).

**Table 1:** Overview of the carcasses and sites included in the study.

Site	GPS	Age class, gender, body weight of the carcasses	Date of Date of placement of the carcasses	End of monitoring	Forest stand type and altitude
Onomyšl (I)	N 49°55.34427′ E 15°6.65680’	piglet ♂ 36 kg yearling ♂ 48 kg adult ♀ 73 kg piglet ♂ 23 kg	11 JAN 2019 03 MAY 2019 07 AUG 2019 02 NOV 2019	28 APR 2019 05 AUG 2019 07 NOV 2019 06 FEB 2020	*Picea abies* and *Pinus sylvestris*, 30 years; 414 m a.s.l.
Kostelec nad Černými lesy (II)	N 49°56.92620′ E 14°54.36413’	adult ♀ 74 kg adult ♀ 82 kg yearling ♂ 63 kg piglet ♀ 18 kg	19 JAN 2019 03 MAY 2019 30 JUL 2019 13 NOV 2019	21 MAY 2019 06 AUG 2019 08 SEP 2019 04 JAN 2020	*Picea abies*, 40 years; 443 m a.s.l.
Slapy – Buš (III)	N 49°47.43740′ E 14°24.28262’	adult ♂ 68 kg yearling ♂46 kg yearling ♀ 62 kg piglet ♀ 20 kg	22 JAN 2019 11 MAY 2019 6 AUG 2019 4 NOV 2019	21 JUN 2019 13 JUL 2019 XXXXXXX 19 JAN 2020	*Betula pendula*, 20 years; 358 m a.s.l.
Drahany (IV)	N 49°27.01468′ E 16°48.58247’	piglet ♂ 43 kg yearling ♂ 52 kg yearling ♀ 71 kg piglet ♂ 20 kg	15 JAN 2019 10 MAY 2019 02 AUG 2019 01 NOV 2019	13 APR 2019 05 AUG 2019 23 SEP 2019 18 NOV 2019	*Picea abies*, 40 years; 614 m a.s.l.
Loket (V)	N 50°11.40495′ E 12°46.78647’	piglet ♀ 38 kg adult ♂103 kg piglet ♀ 17 kg piglet ♀ 22 kg	06 FEB 2019 02 MAY 2019 01 AUG 2019 06 NOV 2019	23 MAR 2019 10 AUG 2019 08 SEP 2019 14 DEC 2019	*Picea abies*, 100 years with natural regeneration; 626 m a.s.l.
Podveky (VI)	N 49°50.17067′ E 14°59.70358’	piglet ♀ 38 kg yearling ♀ 55 kg yearling ♂ 53 kg piglet ♂ 19 kg	18 JAN 2019 01 MAY 2019 29 JUL 2019 30 SEP 2019	26 MAY 2019 01 AUG 2019 30 OCT 2019 30 DEC 2019	*Picea abies*, 15 years; 452 m a.s.l.
Zalíbená (VII)	N 49°48.88495′ E 14°58.77662’	piglet ♂ 45 kg yearling ♂ 57 kg yearling ♀ 52 kg yearling ♀ 56 kg	18 JAN 2019 06 MAY 2019 01 JUL 2019 03 OCT 2019	02 MAY 2019 09 JUN 2019 29 SEP 2019 28 DEC 2019	*Picea abies*, 30 years; 418 m a.s.l.

The camera trap was stolen in the case of location III (Autumn season).

The individual studied forest stands were selected before the study using the GIS environment according to the age of young forest stands. The young forests stands (< 40 years), mainly composed by coniferous tree species (especially by Norway spruce), represent the primary habitats where the ASF-infected wild boar carcasses and deathbed patches were found in the Czech outbreak in 2017 (31). Similar results were confirmed also by a recent study in Lithuania, where ASF-infected wild boars sought shelter in quiet areas (34), which corresponds to conditions of coniferous stands. Therefore, those forest stands are key deathbed patches of ASF-infected individuals in the real outbreak. For the carcass attractiveness evaluation, seven wild boar carcasses were placed in seven preselected sites during every season (i.e., winter, spring, summer, and autumn) during the monitored study period from January 2019 to February 2020, which means in total 28 carcasses were used The control locations were randomly selected empty spaces within 200 meters form the carcass in the same forest stands to avoid confounding effects of environmental conditions (e.g., altitude, vegetation cover, tree species composition, local and landscape habitat structure). Similarly, control locations were placed in the field at the same time as the cameras which monitored the carcasses. To ensure comparable, slower decomposition of the carcasses, all wild boars were hunted and killed by a single head shot following Czech legislative regulations. The carcass data, such as sex, age class, weight, and placement date, are listed in Table 1.

The wild boar presence and activity on study and control sites were monitored by camera traps UOVision UV 595 HD with a resolution of 12 megapixels, HD video (1,080 P), and trigger speed of 0.65 s.^1^ The game cameras were installed on a selected tree at a distance of 4 to 8 meters from the carcass. Cameras were set in video mode with automatic recording of the date and time of the wild boar visit. The video length was set to 30 s with a window of 1 min between recordings. The carcasses and cameras were inspected every 2 weeks to check the carcass status and battery charge. The monitoring was completed when all edible biomass of the carcass was consumed or removed by scavengers or wild boar, and no evidence of the carcass was on the monitored plots. All video sequences with wild boar presence were analyzed from the aspect of number, sex, and approximate age of individuals (i.e., adult male, adult female, unspecified adult, subadult, and piglet). For each recording, we evaluated additional parameters such as duration of carcass setting (in days), and time duration from sunrise and sunset. Sunrise and sunset data were obtained from the web source Sunrise Sunset^2^ for each location. As the main aim of the article was to highlight the attractiveness of carcass, we recorded only the presence of wild boar without conducting a deeper analysis of behavior. In addition, the percentage of direct contact between the wild boar and the carcass was evaluated, considering only instances where physical contact between the wild boar and the carcass was recorded.

### Statistical analyses

The analyses were separated into four parts: analysis of the number of wild boar recordings, sex-age proportions, wild boar detection time, and the time span before the first contact with carcass.

Regarding the analysis of the number of wild boar recordings, basic summary statistics were computed to provide a general overview of collected data. Subsequently, analysis of the number of detected individuals for each study location and season was conducted, and these data were statistically compared between locations where the carcass was placed in comparison to control locations using paired-sample Wilcoxon rank-sum test (35). Non-parametric statistics were selected because the assumption of normality, tested by the Shapiro–Wilk test was violated (36).

Chi-squared test was used to analyze whether the distribution of detected individuals across sex-age categories depended on the type of location (carcass site vs. control site), conducting the analysis separately for each season. The times of wild boar detections were analyzed in relation to sunrise and sunset on the current day. We analyzed the recorded time difference to sunrise/sunset to eliminate the effect of the day length in different seasons. For each recording, the time difference from sunrise/sunset was computed (depending on which was closer to the time of detection), and those values were compared between locations with carcass and control in every season. For such comparison between carcass and control locality, the Wilcoxon rank-sum test was used separately for each season (the assumption of data normality, tested by the Shapiro–Wilk test, was violated in some cases). We used the Levene test (37) to assess whether the variances in time differences from sunrise or sunset were significantly different between carcass locations and control locations. The time of recording relating to sunrise/sunset was also analyzed via circular statistics. Besides the visual representation of the numbers of detected wild boar in the carcass and control locations, we have specifically tested for “uniformity” of observations (i.e., whether the observations were evenly distributed across all hours) via the Rayleigh Test (38) and for the differences between the time of detection of individuals in relation to sunrise/sunset for the carcass and control locations via the Watson-Williams Test (39). We have divided time data into an hour scale for this analysis.

Lastly, the analysis of the time duration in days from carcass and photo-trap setting to the first recorded activity of wild boar was performed. Besides basic statistics and graphical representation of data, the paired-sample Wilcoxon rank-sum test was used for testing for differences between carcass and control locations.

All statistical procedures were performed using R software (40) at a confidence level alpha = 0.05. Wilcoxon-rank sum test and Shapiro–Wilk normality test were conducted via functions in package “stats,” which is integrated to R distribution. Package “car” (41) was used for performing Levene test and package “circular” (42) for Rayleigh test and Watson-Williams test.

## Results

**Figure 1:**
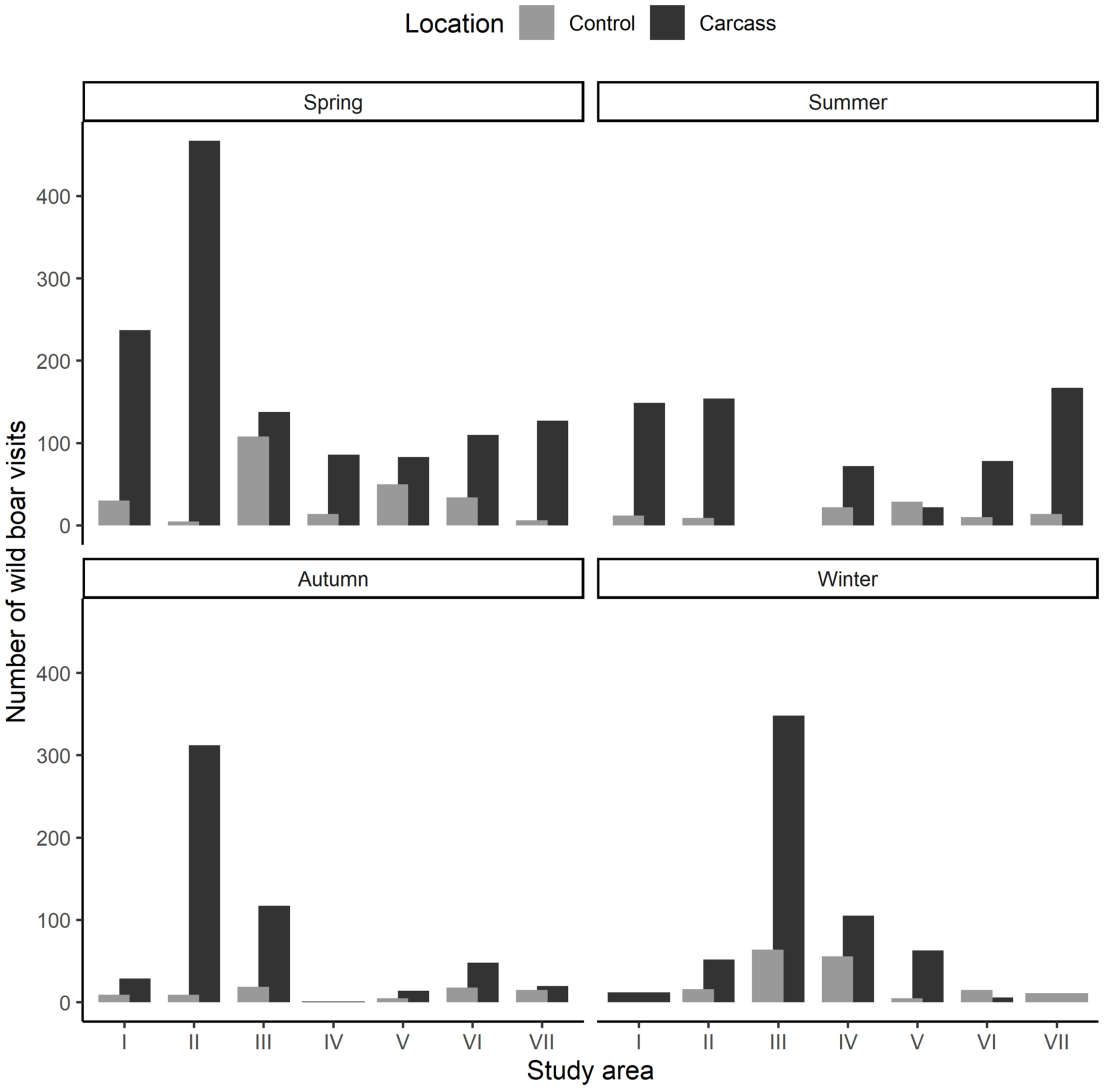
Number of detected wild boar in particular study locations (I–VII) with a carcass and control sites in different seasons.

The overall comparison of numbers of wild boar visits in the carcass and control locations showed significant results (data for each study area in each season were compared; paired-sample Wilcoxon rank-sum test, V = 5.5, *p* < 0.001; Figure 1). In particular, the number of recordings of wild boar in locations with a carcass (3,017 records during 1,156 visits, i.e., mean group size of 2.61 individuals) were 5.2 higher than on control locations (585 records during 242 visits, i.e., mean group size of 2.42 individuals), which suggest an extreme level of attractiveness of wild boar to the carcass. At locations with carcass, 49.9% of visits were of one individual, 17.3% of two individuals and the rest (32.8%) in group consisting of more than two individuals. The largest recorded group consisted of 19 individuals. For control locations, 58.3% of visits were of one individual, 12.0% of two individuals and the rest (29.7%) in group consisting of more than two individuals. The largest recorded group also consisted of 19 individuals. We observed significant seasonal differences in visit frequency between carcass and control locations. Specifically, in spring, there were 1,248 visits in locations with a carcass compared to 247 at control locations, indicating 5.05 times more visits to the carcass. In summer, locations with a carcass received 642 recordings, while the control locations had 96, reflecting a 6.7-fold increase. In autumn, locations with a carcass had 541 recordings compared to 75 at the control, showing 7.2 times more visits. Finally, in winter, there were 586 recordings in locations with a carcass versus 167 at the control, representing a 3.5-fold increase (see Figure 1).

Moreover, the analysis of wild boar visits to carcass sites focused on evaluating the percentage of direct contact between wild boars and the carcasses. The percentage of direct contact varied by season. In autumn, direct contacts were observed in 340 out of 541 visits (62.8%); in spring, in 889 out of 1,248 visits (71.2%); and in summer, in 478 out of 642 visits (74.5%). The highest percentage of direct contacts occurred in winter, with 493 out of 586 visits (84.1%).

From the total of 3,602 detected wild boar visits, we were able to determine the age of individuals in the case of adults, as well as the sex for 3,437 individuals (95%). The other 165 individuals were recognized as adults without further sex specification (5%). The most frequent category was piglets, with 1,817 recordings (50%), followed by subadults (942 recordings, 26%), adult females (509 individuals, 14%), and adult males (169 individuals, 5%).

In spring, only 8% (17 individuals) of all detected subadults were identified at the control location, and other subadult individuals (203 individuals) were recorded at location with a carcass. For additional sex-age categories, the difference was not as pronounced: adult females (31 individuals, i.e., 13% at the control location, 203 individuals at the location with a carcass), piglets (173 individuals, i.e., 19% at the control location, 730 individuals at the location with a carcass), and adult males (13 individuals, i.e., 24% at the control location, 41 individuals at the location with a carcass). The chi-squared test indicated a significant association between the sex-age categories and number of detections per location type (carcass vs. control) during spring (chi-squared = 20.87, df = 3, *p* < 0.001).

A similar trend was observed in the summer: only 5% of subadults were observed at the control locations (7 vs. 141 individuals at the location with a carcass), followed by adult females (11 vs. 106 individuals, 9%), piglets (66 vs. 362 individuals, 15%), and adult males (10 vs. 21 individuals, 32%) as in the previous example. For summer, the chi-squared test also indicated a significant association between the sex-age categories and number of detections per location type (carcass vs. control, chi-squared = 22.70, df = 3, *p* < 0.001).

No significant differences in ratios of detected individuals divided by sex-age categories and location were found in the autumn. The numbers of individuals detected at control locations were as follows: 10% for subadults (21 vs. 217 individuals), 8% for adult females (7 vs. 77 individuals), 12% for adult males (5 vs. 36 individuals), and 15% for piglets (32 vs. 177 individuals). The chi-squared test did not indicate a significant association between the sex-age categories and number of detections per location type (carcass vs. control) during autumn (chi-squared = 5.55, df = 3, *p* = 0.14).

In winter, the ratios of detected individuals at the control locations were higher than in other seasons. The lowest ratio was found for piglets (15%, 41 vs. 236 individuals), followed by subadults (26%, 86 vs. 250 individuals), adult females (32%, 24 vs. 50 individuals), and adult males (33%, 14 vs. 29 individuals). Similarly as for spring and summer, the chi-squared test indicated a significant association between the sex-age categories and number of detections per location type (carcass vs. control, chi-squared = 17.88, df = 3, *p* < 0.001).

**Figure 2:**
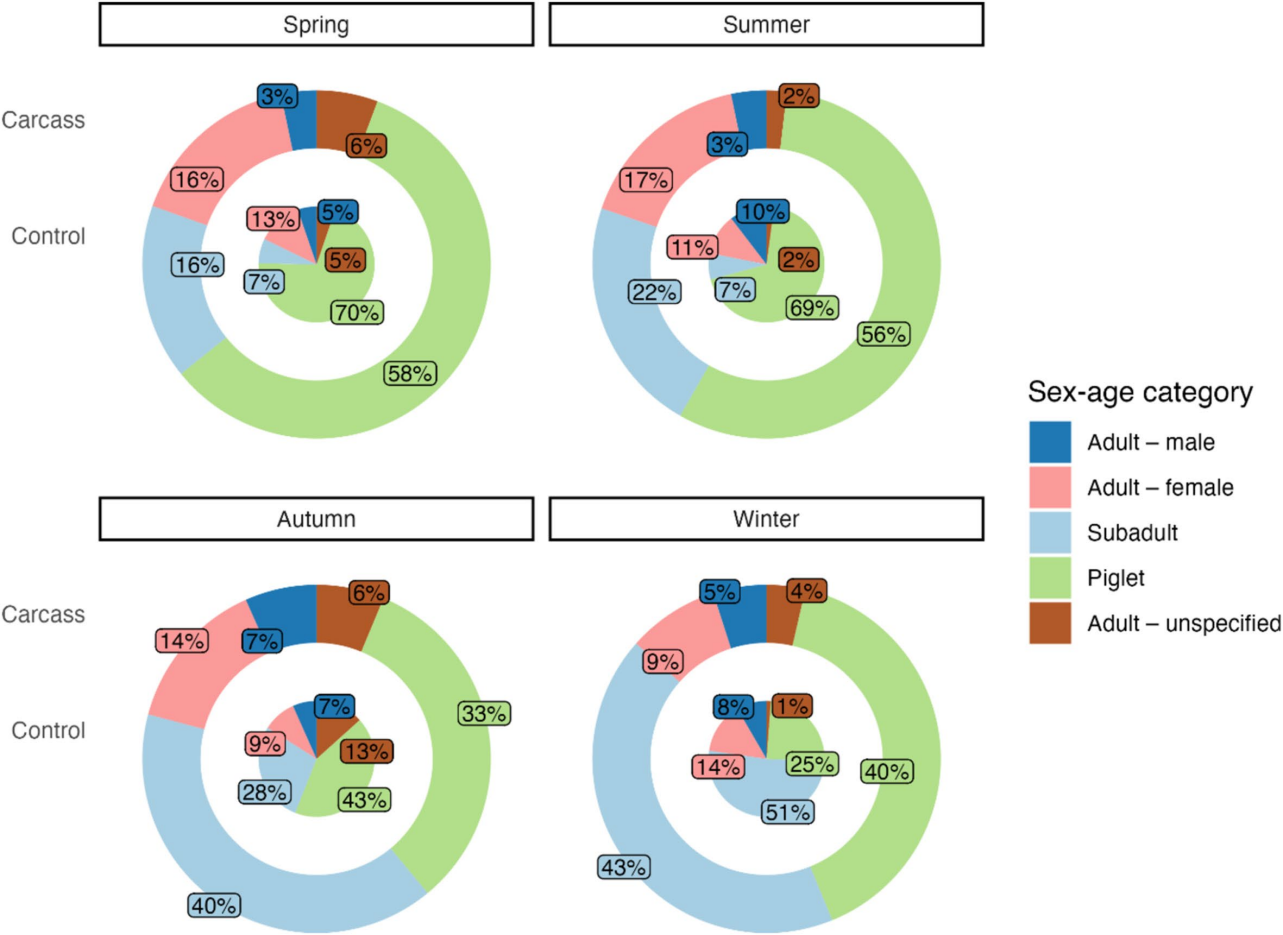
The proportion of sex-age categories in locations with a carcass and control locations for different seasons. The outer circle shows the sex-age categories in a location with a carcass, and the inner circle represents the control location.

Relative frequencies of individuals divided into sex-age for each season are shown in Figure 2.

In spring, recordings at the carcass location were obtained significantly sooner before sunrise compared to the control location (mean: 1.49 h before sunrise for the carcass location and 0.88 h before sunrise for the control location, *p* = 0.006). Comparisons for other seasons were not significant. Obtained *p*-values are as follows: summer—*p* = 0.61, autumn—*p* = 0.24, winter—*p* = 0.12. Mean difference values from sunrise in hours are negative in all cases, i.e., the majority of wild boar were recorded before sunrise. The mean hour differences for seasons with insignificant differences are as follows: summer—3.18 h before sunrise for the location with a carcass, 2.67 h before sunrise for control locations, autumn—4.60 and 4.18 h, and winter—2.31 and 2.04 h.

In the case of the time difference of recordings from sunset, significant differences were observed between control locations and locations with a carcass in both spring and summer (*p* < 0.001 in both cases, with recordings at the carcass locations occurring later). The mean time differences to sunset for each season are as follows: spring—0.85 h after sunset for the carcass locations and 1.10 h before sunset for the control locations; summer—1.98 h after sunset for the carcass locations and 0.90 h after sunset for the control locations; autumn—5.43 h after sunset for the carcass locations and 5.60 h after sunset for the control locations; and winter—1.70 h after sunset for the carcass locations and 2.16 h for the control locations.

**Figure 3:**
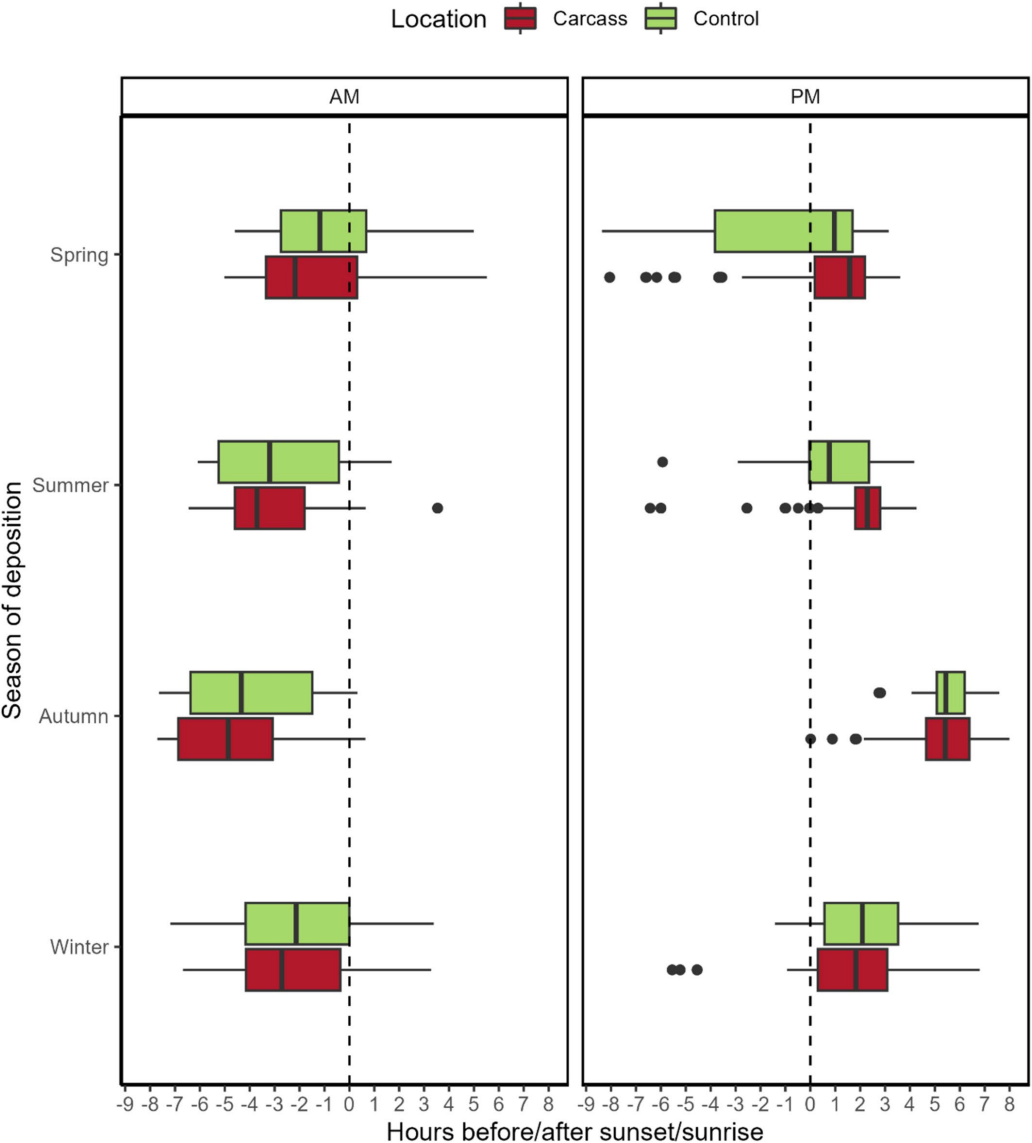
Detection times of wild boar in locations with a carcass and control related to sunset/sunrise. Sunset/sunrise is depicted by a dashed line in the plot. The plot is divided into two parts—AM, for sunrise, and PM, for sunset. The dots indicate outliers for respective variants.

We also tested variations in variances between wild boar recordings to sunrise and sunset at the carcass and control locations. For sunrise, significant differences were observed in all seasons except for spring—*p*-values: spring—*p* = 0.74, summer—*p* < 0.001, autumn—*p* = 0.03, and winter—*p* = 0.005. Variation was consistently higher at the control locations for significant results. For sunset data, the results were the opposite; the only significant result was obtained for spring (p < 0.001, with variation for the control location again being higher). Other p-values are as follows: summer—*p* = 0.25, autumn—*p* = 0.24, and winter—*p* = 0.78. For a graphical depiction of the results, see Figure 3.

**Figure 4:**
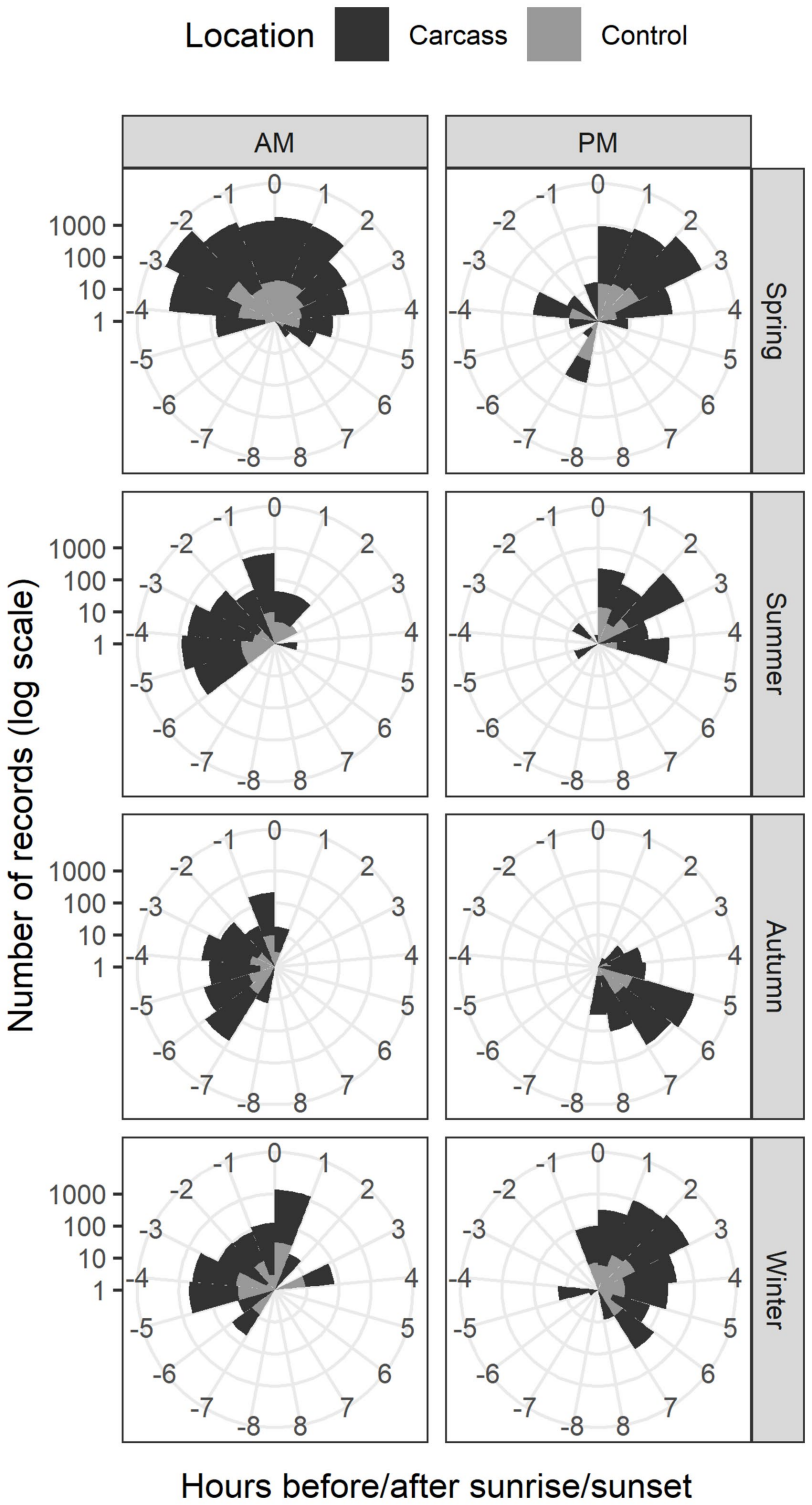
Circular plots for the location with the carcass and control location. Y axis is represented on log-scale due to significant differences between the number of recordings for the location with the carcass and control location.

Circular statistical analyses revealed that the timing of wild boar recordings does not follow a uniform distribution (see Figure 4). We conducted four separate Watson tests to analyze the differences in the number of recordings detected in each hour (i.e., the uniformity of observations between hours, as described above) for each combination of carcass and control locations with sunrise and sunset. These tests showed significant results in all cases (the numbers of detections per hour significantly differed from uniform distribution, *p* < 0.01 in all cases). We further analyzed the data by splitting it into AM (before and after sunrise) and PM (before and after sunset) hours. For both time periods separately, we tested whether there were differences in the distribution of recordings across hours between carcass and control locations. The results showed significant differences in the distribution of observations between carcass and control locations across all seasons (*p* < 0.001 for both AM and PM hours). For example, in carcass locations, animals were often recorded in times, when no animals or only small numbers of them were detected in control locations (animals were observed in broader time window around sunrise/sunset in carcass locations), e.g., −5 and +6 h around sunrise in spring or −6 and −3 h before sunset in summer.

**Figure 5:**
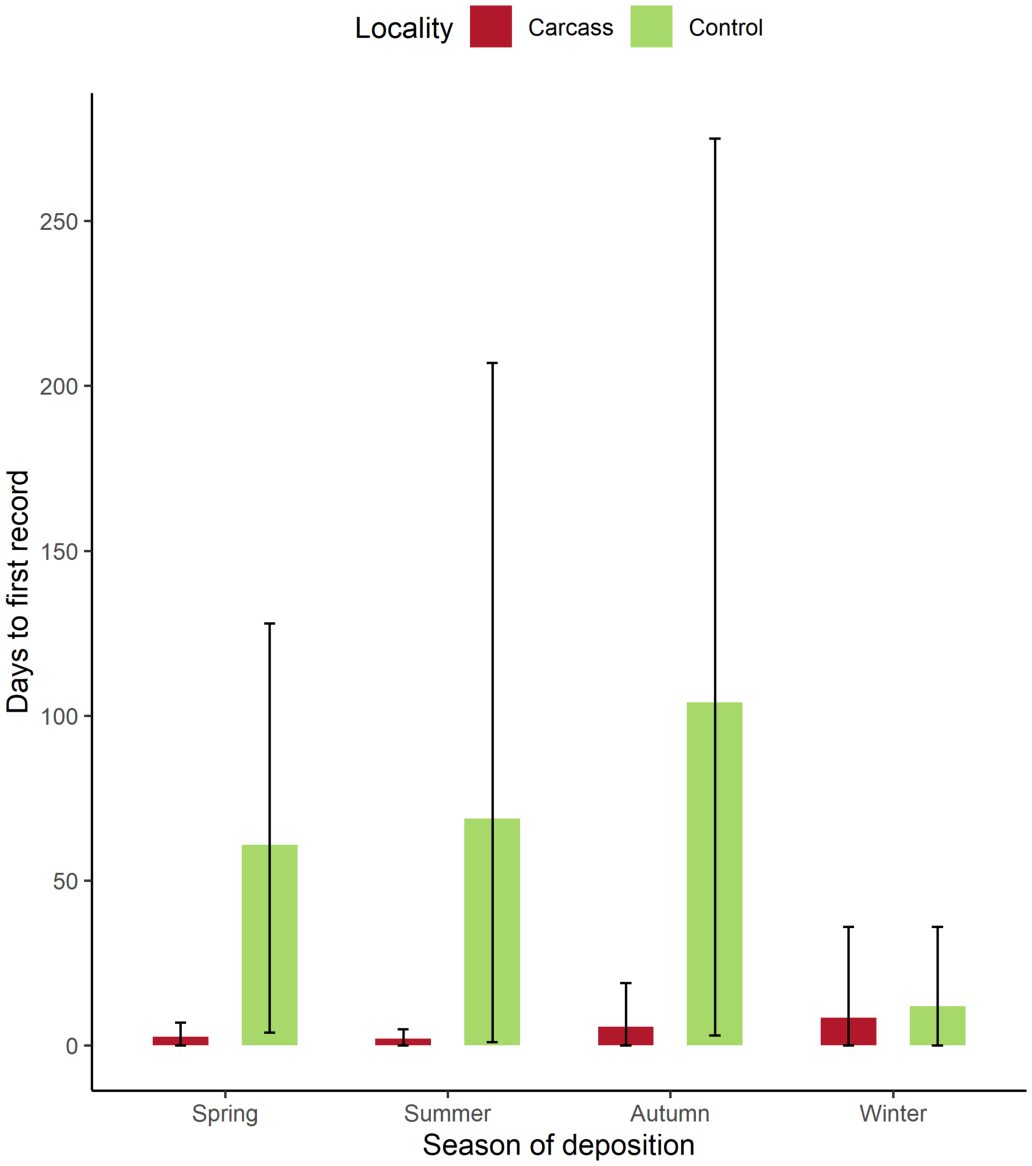
Number of days to the first recording of wild boar activity on control locations and locations with the carcass. Bars stand for mean values, whiskers for min and max values for each season, and location type.

The analysis of the time of the first visit to the carcass showed that wild boar found the carcass in a relatively short time (Figure 5). The average values amongst all locations were around 2 days in spring and summer, around 6 days in autumn, and 8 days in winter. Also, during spring and summer, the maximum recorded times to find the dead body were 7 and 5 days in particular locations. In autumn, the maximum days needed to find the dead body were 19 days, and in winter, up to 36 days. Nevertheless, in all seasons, wild boar were able in some locations to find the dead body on the same day it was placed were observed. The comparison of days between the date of carcass setting and the first recording of wild boar activity showed significant results (Wilcoxon paired-sample test, *p* = 0.03). On control locations, the first wild boar detection was found after a much longer period compared to locations with the carcass (spring—2.6 days on average for the carcass location vs. 61 days on average for control locations, summer—2 vs. 69 days, autumn—5.7 vs. 104 days, and winter—8.4 vs. 11.8 days), although very high variance was observed for all seasons. Minimum values for control locations were 0 days for winter and 4 days for spring, while maximal values were 36 days for winter, followed by 128 days for spring, 207 days for summer, and 275 days for autumn. The average values through the year were 4.7 days to the first visit to the carcass location and 61.5 for the control.

## Discussion

African swine fever transmission is driven by several factors that are changing across the geographic conditions where the virus is present, both in wild boar and domestic pigs’ populations (9). In Europe, it seems that the infected carcasses play the most crucial role in transmission (30, 31, 43, 44) besides the human factor, which transports the virus long distances, for hundreds of kilometers, mostly through pork products (45). Therefore, it is necessary to understand all aspects of wild boar behavior toward the carcasses of its own species, about which we still have limited information. However, data on carcass attractiveness can only be compared to the general knowledge of wild boar activity within their home range, as previous studies have investigated wild boar interactions with carcasses (20, 21) but have not included a comparison with a control location, as was done in this study. One of the main ways to compare and express carcass attractiveness is by comparing the number of wild boar visits to the control location in comparable conditions, which was over five times higher throughout the year. Based on those findings, it is apparent that the carcass is perceived by wild boar as an attractant. The highest difference in the number of visits in location with carcass and control location was found in the spring and summer seasons. During the warmer period, the wild boar activity around the carcass was greater compared to the control location. This can be explained by the rapid carcass decomposition by scavenging insects, which is followed by a strong odor of decaying carcasses (46) and therefore, carcasses could be more easily detected.

In general, we have detected 3,602 wild boar visits for carcass and control locations combined, from which the sex and age could be determined in 95% of the visits. Not surprisingly, piglets were detected in most of the cases, which corresponds to normal wild boar population structure and high litter size per adult female (47). In our case, the proportion of piglet detection exceeded 50% of recordings in the spring and summer periods in the carcass location, with a decreasing tendency for autumn and winter. A similar trend was also found in the control locations. It can be explained by hunting pressure followed by decreasing piglet proportion through the season (48). Moreover, wild boar has enough fodder opportunities in a fragmented landscape of high-energy crops throughout most of the year (49, 50), and therefore, the body mass and appearance soon resemble subadults more than piglets. Interestingly, subadults were recorded in significantly higher numbers at the carcass location compared to the control site, particularly in spring. This pattern may be due to reduced natural food availability during this period. Most natural food sources, such as beechnuts, acorns, and other tree seeds, have already been consumed by winter, and cereal crops are not yet fully grown. Therefore, the decomposing carcass could be considered a food source by wild boars, as chewing on bare ribs (especially in summer) was confirmed in a previous study by Probst et al. (20). Also, during this period, subadult males are excluded from family groups as adult females focus on raising the new piglet generation. This makes it more challenging for subadults to find food opportunities, prompting them to intensify their search, which leads to more frequent encounters with carcasses.

From the ASF transmission point of view, it is important to highlight that there is an explicit assumption that the individuals, due to the fluctuating age distribution (according to camera trap monitoring), are from different groups and simultaneously visited the same carcass. Additionally, the wild boar social structure is important in the context of ASF transmission. At the social network level, young animals up to 2 years of age showed greater between-group connectivity than adult ones (51) and therefore, the observed structure of monitored individuals indicates a higher risk especially during the spring and summer seasons, due to the high proportion of piglets in the population. These facts allow us to observe how quickly ASF can spread during out-group interactions.

The time of detected wild boar activity was another aspect of behavior that was analyzed. In common circumstances, the diurnal activity usually involves movement between resting areas and feeding sites (64). The highest proportion of wild boar active behavior occurs around midnight and morning hours (65, 66). In this study, the wild boar activity was recorded especially close to sunset/sunrise during most of the year. The earliest visits around sunset were found in spring, when the decomposition process is relatively fast, which is characterized by a strong odor (43) (mentioned earlier). However, the distribution of wild boar visits in relation to the time before and after sunrise and sunset was not uniform across seasons. Despite the significant differences observed across different seasons, no clear temporal pattern was identified throughout the entire year. These findings suggest that the temporal distribution of wild boar activity is heavily influenced by resource availability and sensory cues, such as odor intensity, at carcass sites. The lack of a uniform temporal pattern throughout the year may reflect the interplay between environmental conditions, seasonal variations in decomposition rates, and the foraging strategies of wild boar. Interpreting these differences emphasizes the role of carcass sites as focal points for temporally clustered activity, contrasting with the more dispersed activity observed in control locations.

The greatest differences between the location with the carcass compared to the control site were found at the time of the first detection of wild boar on the camera trap. On average, the first wild boar was detected after 4.7 days in the carcass location and after 61.5 days in the control site, with the highest average difference found in autumn (5.7 vs. 104 days). The number of wild boar recordings between the carcass and control locations during different seasons could be caused by variations in wild boar population density throughout locations and by the home range size changes. Wild boar shows remarkable intraspecific variations in home ranges across various habitats. Annual home range size varies between 400 ha to 6,000 ha, with an average size of around 800 ha (52, 53). According to wild boar home range size, ASF is spreading gradually at a steady pace of 1.5 km per month throughout the year (54). The larger home range sizes were confirmed in the autumn and winter periods (55), which is influenced by several factors, e.g., by the rut season where the wild boar has higher daily home range sizes compared to the rest of the year (56). Another aspect can be the rebalance caused by the autumn hunting season, which also affects the home range size and the wild boar activity and space use (57). Moreover, the habitat preference of wild boar is driven by food source availability. In the late summer, the standard behavior patterns and habitat utilization of wild boar can be disrupted and changed by the crop harvest. In forested areas, the habitat preference is affected by oak species (*Quercus* spp.) and European beech (*Fagus sylvatica* L.), especially in the mast years of the aforementioned deciduous trees (47, 49, 58). This means that if the wild boar’s basic life needs are satisfied, it does not make much sense for them to move over greater distances. On the contrary, most of the carcass and control sites in our study were in Norway spruce forests with low availability of natural food sources for wild boar, which may explain the later visits after sunset during autumn. It was previously proven that in poor nutritional conditions, wild boars move more in search of food and water, increasing their home range (56, 59). Moreover, wild boar behavior and the time of the carcass visits can be significantly affected by supplementary feeding provided by hunters. This means that the movement of wild boars is influenced by the number and location of feeding places in their natural habitat. The amount of supplemental food can be approximately 1,000 kg per year per 100 ha in particular locations, and the feeding is targeted primarily in the autumn and winter periods (52).

Therefore, it appears that the high risk of ASF transmission through infected carcasses is prevalent throughout the year. The potential ASF transmission is affected by subsequent wild boar movement after contact with the infected carcass. The daily distances traveled by wild boar are usually between 10 to 20 km (53, 60, 61). However, if the area lacks suitable food sources, the wild boar is forced to increase the distances traveled, which increases the risk of spreading ASF. On the other hand, if there is sufficient food, water, and shelter, most young wild boar (70–80%) do not disperse further than 5 km from their natal ranges (62, 63). All sex-age wild boar categories occasionally move long distances of 50–250 km in a straight line in rare situations (62, 63), and in this example, wild boar can walk 30–40 km within 24 h and 200–300 km in 10–15 days (61).

## Conclusion

Thus far, it has not been determined whether the wild boar carcasses are visited purposefully or whether they are visited as part of the habitual movement of wild boar in the location. The answer to this question is made clear by the conclusions presented in this study, which confirmed the immense attractiveness of the carcass for the wild boar population across the seasons. Based on the visit differences between locations with the carcass and the control in a comparable habitat, it is evident how attractive the wild boar carcass is to their own species, which has confirmed a critical role in the ASF transmission. Our results confirm important implications for the understanding of ASF spreading among individuals of wild boar populations. We clearly demonstrated that carcasses of wild boars are highly attractive for wild boars during the different seasons, which pose a high risk of ASF transmission throughout the year. Therefore, there is an urgent need for early detection and removal of infected carcasses from the environment. This seems to be particularly urgent in spring and autumn months, when wild boars detect carcass earlier than in the rest of the year.

## Data Availability

The raw data supporting the conclusions of this article will be made available by the authors, without undue reservation.

## Footnotes

^1^ www.uovision.com

^2^ https://api.sunrise-sunset.org/
